# Ultrasound-detected synovitis in symptomatic hand osteoarthritis is associated with functional impairment: data from the LIHOA cohort

**DOI:** 10.1186/s42358-025-00513-z

**Published:** 2026-01-31

**Authors:** Francisco Vileimar Andrade de Azevedo, Antonio Matos de Souza Filho, Ana Carolina Matias Dinelly Pinto, Guilherme Ferreira Maciel da Silva, Artur Nascimento da Rocha, José Carlos Godeiro Costa Junior, Cláudio Régis Sampaio Silveira, Francisco Airton Castro Rocha

**Affiliations:** 1 Postgraduate Program in Medical Sciences, Federal University of Ceará, Fortaleza, CE Brazil; 2 Department of Internal Medicine, Faculdade de Medicina, Universidade Federal do Ceará, Fortaleza, CE Brazil; 3 Centro Pasteur Fiocruz de Imunologia e Imunoterapia -Fiocruz CE, Fortaleza, CE Brazil; 4 Instituto São Carlos de Ensino e Pesquisa/São Carlos Imagem, Fortaleza, CE Brazil; 5 Instituto de Biomedicina – Laboratório de Investigação em Osteoartropatias, Rua Coronel Nunes de Melo, 1315 -1º. Andar Rodolfo Teofilo, Fortaleza – CE CEP, 60430-270 Brazil

**Keywords:** Osteoarthritis, Hand osteoarthritis, Ultrasound, Diagnostic imaging

## Abstract

**Aim:**

To determine the association of ultrasound (US) parameters with clinical features of patients with symptomatic hand osteoarthritis (HOA).

**Methods:**

72 patients (61.9 ± 10.3 years-old), 94 (92%) women with a diagnosis of painful HOA fulfilling ACR criteria seen between August 2019 and May 2023 were evaluated. Evaluations included pain (0–10 cm; VAS, visual analogue scale), grip (GrS) and pinch (PiS) strength (KgF), Cochin hand functional scale (CHFS), functional index for hand osteoarthritis (FIHOA), number of interphalangeal joints (IP) with pain/nodes, and serum C reactive protein (CRP). US was semi-quantitatively scored (0–3) in the most painful IP, assessing grey scale synovitis (GSS), synovial thickening (ST), effusion, and power Doppler signal (PD). Comparisons were made assuming the variables as independent and continuous, using Student’s “t” test and multivariate analysis.

**Results:**

GSS ≥ 2 synovitis was associated with lower GrS (*p* = 0.022) with a non-significant trend to be associated with higher FIHOA (*p* = 0.074) and Cochin (*p* = 0.093) scores. ST was also associated with lower GrS (0.003) and higher FIHOA (*p* = 0.045) with also a trend for higher Cochin score (*p* = 0.068). All but 4 joints had a negative PD, which precluded further evaluation. US parameters were neither associated with pain at rest/movement nor with number of IP nodes. Also, US features were similar regardless of serum CRP level and presence of OA in joints other than the hands.

**Conclusions:**

GSS and ST are associated with worse physical function, meaning lower GrS, but not with pain level in symptomatic HOA patients.

## Background

Osteoarthritis of the hand (HOA) is a highly prevalent chronic inflammatory disease that is associated with pain and disability, particularly in the elderly [1–3]. Inflammatory changes happening in the joints of patients with HOA are linked to progression of structural damage [4–8]. In addition to diverse presentations concerning symptoms and course of the disease, HOA heterogeneity can also be distinguished by erosive, nodal interphalangeal (IP) HOA and thumb-based HOA subsets or phenotypes [9, 10]. Besides the lack of association between clinical severity and radiological damage, radiography findings in IP HOA are neither sensitive to change nor are alterations in the surrounding soft tissue identified [11–13]. Moreover, precise specific biomarkers to classify these phenotypes are still an unmet need in HOA, an issue that impacts evaluating the efficacy of treatment interventions [10, 14]. The ability of ultrasound (US) to detect synovitis, joint fluid, osteophytes, and erosions in addition to excellent availability and low cost turns US an interesting imaging biomarker in HOA [15].

Similar to other diseases, HOA may also be impacted by socioeconomic issues. In this regard, low-income individuals may have additional hurdles to overcome to treat their HOA [16]. Usually, people living with social disadvantages more commonly have work that can be classified as blue-collar jobs, meaning more need for manual activities, which has been linked to a higher disease burden [16, 17]. Also, less access to specialized care may retard diagnosis as well as treatment, which may impose another difficulty to manage this disease.

There is still a lack of studies to support widespread use of US to evaluate HOA and, to our knowledge, there are no studies conducted in low-income cohorts. We report US data captured in patients of our low income HOA (LIHOA) cohort with symptomatic IP involvement, trying to associate US changes with clinical parameters besides focusing socioeconomic issues [16].

## Methods

### Study design and setting

The current analyses include cross-sectional data from the baseline examination of the low income HOA (LIHOA) study [16]. LIHOA is a single-center study of patients with symptomatic radiographic HOA. Patients were interviewed to collect demographic and clinical data, followed by a clinical and radiological assessment of their hands. This study complied with current guidelines for good clinical practice and was approved by the ethics committee of the Universidade Federal do Ceará (CAAE: 02879318.8.1001.5054).

#### Inclusion criteria

Individuals ≥ 40 years-old with nodal symptomatic HOA according to the American College of Rheumatology (ACR) criteria [18] with ≥ 2 symptomatic joints among proximal/distal IP joints or 1st IP joint and radiographic Kellgren–Lawrence (KL) stage ≥ 2. Symptoms of HOA was defined by the presence of pain plus stiffness or aching in an IP joint on at least half of the days in the last 4 weeks.

#### Exclusion criteria

diagnosis of inflammatory arthritis particularly rheumatoid and/or psoriatic arthritis; gout or calcium dihydrate pyrophosphate disease; infections or acute trauma to the hands; hereditary hemochromatosis; carpal tunnel syndrome, De Quervain’s tendinopathy, Dupuytren’s disease, diabetic neuropathy, thoracic outlet syndrome, previous upper limb surgery, and fibromyalgia.

### Clinical and laboratory data

A case report form was used to register all clinical data including disease (HOA) duration, presence of continuous chronic pain in hand joints, and the number of pain flares occurring over a six-month period prior to study entry considering continuous if pain was reported in ≥ 50% of the days or by flares if reported in < 50% of the days during the 6 months period of observation. Pain in the joints of the hands (at rest, activity, and upon palpation), presence of soft tissue swelling (i.e., capsulo-synovial enlargement), most painful joint in both hands using a 0–10 cm visual analogue scale (VAS), as well as the number of distal and proximal IP Heberden’s and Bouchard’s nodes, respectively, were registered. Pinch (PiS) (pulp pinch) and grip strength (GrS) of the most painful finger and the most symptomatic hand were measured using a Saehan^®^ hydraulic pinch gauge and a hydraulic hand dynamometer, respectively, registering the best result of 3 tests. Patient reported outcomes were assessed using fulfilment of the Portuguese version of FIHOA, the Cochin hand functional disability scale as well as patient self-assessed level of pain during the last 48 h in the most symptomatic joint at rest and activity, using a 0–10 cm VAS [19–21]. Concomitant OA in joints other than the hands (shoulders, cervical and lumbar spine, hips, and knees) was registered based on available clinical and imaging data. Knee OA diagnosis was made using ACR clinical criteria and hallux valgus (bunion) secondary to OA was determined based on clinical history to exclude other causes, physical examination, and presence of a hallux valgus angle ≥ 20° [22, 23]. Clinical examination was done by two senior board-certified rheumatologists (FVAA, FACR). Body mass index and obesity (BMI ≥ 30) were registered. A blood sample was collected for analysis of serum C-reactive protein levels. Ongoing pharmacological treatments for OA were also recorded, including nonsteroidal anti-inflammatory drugs (NSAIDs), opioids, and symptomatic slow acting drugs for OA (SYSADOA), encompassing nutraceuticals and phytocompounds.

### Socioeconomic data

Income evaluation considered monthly family income using March 2022 as reference for conversion of Brazilian R$ to US$ currency, based on monthly minimum wage (MW), as follows: <1, 1≥/<3, ≥ 3 MW, which corresponded to < 300.00 US$, 300.00 ≥/< 900.00 US$, and ≥ 900.00 US$. Families earning < 3 MW were considered as low-income, according to official data. Current or previous occupations were arbitrarily classified as either blue/white collar jobs using the Brazilian classification of occupations [16].

### Imaging data

US examinations of the target hand were performed by an experienced radiologist blinded to clinical data (JCCJr) using a Logic S8 ultrasound machine (General Electric) with a 6–15 MHz linear array probe and a preset for grayscale synovitis (GSS) and power Doppler (PD; pulse repetition frequency 0.6 kHz, frequency 7.7 MHz). The examination was carried out with the patient and operator sitting opposite each other, with the patient’s hands resting on a small table. The ultrasonographer scored the most symptomatic IP joint in both hands: distal (DIP) and proximal (PIP) interphalangeal joints, including the first IP joint. The joints were scanned dorsally, with longitudinal projection, from the radial to the ulnar side. GSS, PD signal, synovial thickening (ST), and joint effusion (JE) were scored using the validated Outcome Measures in Rheumatology (OMERACT) semiquantitative scoring system (0–3) for each component, with higher values ​​indicating more activity. Minor alterations when performing US in HOA may lack clinical relevance. Therefore, GSS ≥ 2, ST ≥ 1 and PD signal ≥ 1 were considered as measures of relevant synovitis [24]. Postero-anterior views of hand radiographs were scored using the Kellgren-Lawrence (K-L) scale by another senior board-certified radiologist blinded to clinical data (CS) registering the maximum score among the 4 distal and proximal IP joints (DIP and PIP, respectively) and the IP of the thumb [25].

### Statistical analysis

Characteristics of the patients were expressed as frequencies and percentages for categorical variables and as means ± standard deviation (SD) or medians (range) and interquartile ranges (IQR) for continuous variables, as appropriate. Comparisons were made using Student’s “t” test, Mann-Whitney, chi-square or simple linear regression, as appropriate, with a significance level of 95% (alpha = 5%). Analysis of data were done using SAS 9.4 M7, SAS Inc.

## Results

### Demographic and clinical characteristics

Among the 119 recruited patients in the LIHOA study, 17 patients were initially excluded, as follows: 6 met exclusion criteria, 6 declined to participate, 3 did not meet radiologic criteria and 2 had no IP nodes. After this stage, of the 102 eligible patients, 30 declined to undergo US examination. Demographic and clinical characteristics of the study population (*n* = 72) are shown in Table 1. Mean age was 60.9 ± 10.6 years-old with 52% participants over 60 years-old, the oldest being 89 years-old. Women were highly predominant (69; 95.8%). Systemic arterial hypertension (43%), metabolic syndrome (40.2%), and dyslipidemia (26.3%) were the most common comorbidities, whereas obesity was present in 19 (26.3%) individuals (Table 1). Mean disease duration was 7.5 ± 7.1 years. Pain on activity was significantly higher than at rest (*p* < 0.001) with patients being mildly to moderately symptomatic on joint assessment at rest and activity, with 3 (3–5) and 8 (5–9) median VAS values, respectively. Fifty-seven (56.4%) patients had ≥ 4 symptomatic joints with a median of 4 (2–8) painful joints at activity. The 2nd distal IP was the most symptomatic (21; 23.3%), followed by the 3rd distal IP joint (19; 21.1%). Most patients had over 4 IP nodes and the majority had OA in joints other than the hands, as follows: 37 (36.2%) in the lumbar and/or cervical spine followed by 28 (29.4%) with knee OA and 21 (20.5%) hallux valgus. Overall functional impairment was considered mild based on a median 8 (5–14) FIHOA value (Table 2).

**Table 1:** Demographic and clinical characteristics of patients in the LIHOA cohort

Variable	Value
Gender	
Female	69 (95.8)
Male	3 (4.2)
Age (years)	60.9 ± 10.6
Current professional situation	
Active	47 (65.2)
Retired	25 (34.8)
Main ocupation (past or current)	
Blue Collar	31 (43)
White Collar	41 (57)
BMI (kg/m²)	27.3 ± 1
Obese (≥ 30)	19 (26.3)
Comorbidities	
Hypertension	31 (43)
Metabolic Syndrome	29 (40.2)
Dyslipidemia	19 (26.3)
Osteoporosis	11 (15.2)
Generalized anxiety disorder	10 (13.8)
Thyroid disease	10 (13.8)
Diabetes mellitus	8 (11.1)
Migraine	7 (9.7)
Depression	4 (5.5)
Cardiovascular disease	3 (4.2)

BMI, Body mass index; HOA, hand osteoarthritis; Blue and white collar jobs refer to jobs associated mostly with manual labor and technical skills and office-based, administrative tasks, respectively. Data represent n(%) or mean ± SD

**Table 2:** Clinical characteristics in the LIHOA cohort

Parameter	Values
Symptom duration (years; mean ± SD)	7.5 ± 7.1
Hand pain intensity at rest (0–10 cm VAS)	3 (3–5)
Hand pain intensity at activity(0–10 cm VAS)	8 (5–9)
Hand pain profile	
Continous pain [n(%)]	55 (66.5)
Evolution by flares [n(%)]	36 (39.5)
IP nodes [median(range)]	8 (6–10)
≤ 3 nodes [n(%)]	11 (11.4)
> 3 nodes [n(%)]	85 (93.7)
Heberden’s nodes [median (range)]	7 (5–8)
Bouchard’s nodes [median; range)]	1 (0–2)
Painful joints at activity [median(range)]	4 (2–8)
Painful joints at palpation [median(range)]	4 (2–8)
Joints with soft tissue swelling [median(range)]	4 (2–8)
Presence of OA at other sites [n(%)]	70 (68.6)
Knee OA [n(%)]	28 (29.4)
Rhizarthrosis [n(%)]	19 (18.6)
Spine [n(%)]	37 (36.2)
Shoulder [n(%)]	9 (8.8)
Halux valgus (Bunion) [n(%)]	21 (20.5)
Grip strengh (kg) (mean ± SD)	14.9 ± 6.6
Pinch strength (kg) (mean ± SD)	1.9 ± 1.2
FIHOA [median(range)]	8 (5–14)
Cochin [median(range)]	19 (6–29)

IP, interphalangeal; OA, osteoarthritis; FIHOA, Functional index for hand osteoarthritis; SD, standard deviation

### Imaging and laboratory data

All but one patient among the 73 participants had the most symptomatic IP joint subjected to US examination, meaning a total of 72 joints examined. The most symptomatic IP joint with synovitis at US was the 2nd distal IP joint followed by the 3rd distal IP. PD signal and joint effusion ≥ 1 were detected in 5.4% and 6.9% of patients, thus preventing performing statistical analysis. The mean total K-L score of the 95 analysed radiographs was 27.6 ± 13.6 with 21 (23%), 38 (41.7%), and 33 (36.2%) patients classified as KL-2, K-L3, and K-L4, respectively. Among the 72 subjected to US evaluation, 39 (54.2%), 26(36.1%), and 7(9.7%) were classified as synovitis grade 1, 2, and 3, respectively. Therefore, there were no patients with absence of synovitis as defined by US scores (grade 0) in the most symptomatic joint among all patients analysed. Laboratory data revealed 0.38 ± 0.22 mg/dL mean ± S.D. serum CRP level with 14 (20%) patients displaying CRP levels above reference value (0.5 mg/dL) (Table 3). Serum CRP levels were 0.43 ± 0.8 mg/dL and 0.32 ± 0.19 in patients with GSS ≥ 2 and GSS < 2, respectively (*p* = 0.2696). Almost 80% of the patients were not taking any specific medications to treat their HOA; SYSADOA prescribed by physicians were used by 13 (12.7%) individuals, comprising 6 (5.8%), 5 (5%), and 2 (1.9%) using hydrolyzed collagen, glucosamine sulfate/chondroitin sulfate (5), and *Harpagophytum procumbens* (1), respectively; oral and topical NSAIDs were being used by 6 (6%) and 3 (3%) patients, respectively; 2 (2%) patients were using 5 mg/d oral prednisone. NSAIDs and prednisone were taken on demand, with no medical prescription. Unfortunately, we were not able to check for dosage, adhesion or combination of these medications. No patient has undergone or was planning to undergo surgery for HOA.

**Table 3:** Imaging and laboratory data in the LIHOA cohort

Parameter	Value
Joints with PD ≥ 1	4 (5.5)
Joints with Effusion ≥ 1	5 (6.9)
Joints with GSS < 2	39(54.2)
Joints with GSS ≥ 2	33 (45.8)
Total K-L score (mean ± SD)	27.6 ± 13.6
N° joints K-L 2	42 (%)
N° joints K-L 3–4	22 (%)
Serum CRP (mean ± SD; mg/dL)	0.38 ± 0.22
Number of patients < 0.5 mg/dL	55 (80)
Number of patients ≥ 0.5 mg/dL	14 (20)

CRP, C reactive protein; GSS, gray scale synovitis; K-L, Kellgren-Lawrence; PD, power Doppler. Data indicate n(%) or mean ± standard deviation

### Associations between US findings and clinical parameters

Due to the infrequent prevalence of grade 3 GSS observed in previous studies, which was consistent with our study, we combined grades 2 and 3 in the analyses. Synovitis meaning GSS ≥ 2 was not significantly associated with pain either at rest (*p* = 0.62; CI -0.79–1.33) or movement (*p* = 0.44; CI -0.54–1.24). Neither the number of joints with swelling (*p* = 0.47; CI -1.0–3.0) or pain (*p* = 0.49; CI -3.76–0.68) on palpation and IP nodes were associated with GSS ≥ 2 score. Similarly, having OA in joints other than those of the hands was not associated with GSS ≥ 2 score (*p* = 0.57; CI -0.37–0.75). Occupation was also not associated with intensity of the synovitis measured by GSS scores, as a chi-square test comparing having a blue or white collar occupation did not reveal an association with GSS scores (*p* = 0.49). Comparison of patients that declared monthly earnings > 3 MW to those earning ≤ 3 MW, using chi-square, also did not reveal differences regarding GSS scores (*p* = 0.81). Serum CRP levels (*p* = 0.6; CI -0.24–0.42), FIHOA (*p* = 0.07; CI -0.25–5.36), and Cochin (*p* = 0.09; CI -0.99 -12.6) scores were also not associated with GSS ≥ 2 score. Notably, a decrease in GrS (*p* = 0.02; CI -6.23–0.048), but not in PiS (*p* = 0.9; CI -0.55–0.49), was significantly associated with GSS ≥ 2 score (Table 4). Similarly, ST was also not associated with pain at rest (*p* = 0.29; CI -0.5- 1.64) or movement (*p* = 0.48; CI -0.58–1.24), presence of IP nodes (*p* = 0.38; CI -0.86–2.22), serum CRP (*p* = 0.46; CI -0.21–0.46), OA in multiple joints (0.53; CI -0.35–0.68) or Cochin score (*p* = 0.068; CI -0.49–13.3) while being significantly associated with higher FIHOA scores (*p* = 0.045; CI 0.06–5.75). Similar to GSS ≥ 2, ST ≥ 2 was significantly associated with reduced GrS (*p* = 0.003; CI -7.19 - -1.48) (Table 5). A simple linear regression revealed a statistically significant inverse relationship between GrS and GSS ≥ 2 [β = 2.97, 95% CI (0.13–5.81), *p* = 0.04] as well as ST ≥ 2 [β = 3.54, 95% CI (0.63–5.46) *p* = 0.018] after adjusting for age, sex, and BMI. On the other hand, the multivariate analysis revealed no statistically significant relationship between PiS and GSS ≥ 2 [β = 0.016, 95% CI (-0.51–0.551), *p* = 0.06] or ST ≥ 2 [β = 0.23253, 95% CI (-0.32–0.78) *p* = 0.40] after adjusting for age, sex, and BMI.

**Table 4:** Impact of gray-scale synovitis in pain and function in the LIHOA cohort

Parameter	GSS < 2	GSS ≥ 2	Mean difference (95% CI)	*P* value
N° painful joints at activity	5.9	4.7	1.60 (-0.59, 3.80)	0.151
Nº painful joints at palpation	8.30	9.13	0.82 (-3.76, 0.68)	0.490
Nº joints soft tissue swelling	6.17	6.81	1.00 (-1.00, 3.00)	0.472
Hand pain at rest (VAS)	3.67	3.94	0.26 (-0.79, 1.33)	0.621
Hand pain at activity (VAS)	7.20	7.55	0.34 (-0.54, 1.24)	0.444
Total number of IP nodes	7.83	8.61	0.78 (-0.73, 2.29)	0.306
Nº other sites with OA	1.1	1.0	0.14 (-0.37, 0.75)	0.578
PiS (kg)	1.83	1.80	-0.03 (-0.55, 0.49)	0.900
GrS (kg)	16.5	13.2	-3.36 (-6.23, -0.48)	0.022
Cochin	18.3	24.1	5.82 (-0.99, 12.6)	0.093
FIHOA	9.06	11.3	2.55 (-0.25, 5.36)	0.074
Serum CRP (mg/dl)	0.33	0.42	0.08 (-0.24, 0.42)	0.602

CRP, C reactive protein; FIHOA, functional index for hand osteoarthritis; GSS, gray-scale synovitis; GrS, Grip strength; IP, interphalangeal; LIHOA, low income hand osteoarthritis; PiS, pinch strength; VAS, visual analogue scale

**Table 5:** Impact of synovial thickening (ST) on ultrasound analysis in the LIHOA cohort

Parameter	ST < 2	ST ≥ 2	Mean difference (95% CI)	*P* value
N° painful joints at activity	5.84	7.18	1.34 (-0.91, 3.59)	0.240
Nº painful joints at palpation	8.27	9.29	1.01 (-1.40, 3.43)	0.406
Nº joints soft tissue swelling	6.09	7.04	0.94 (-1.00, 2.89)	0.336
Hand pain at rest (VAS)	3.57	4.14	0.57 (-0.50, 1.64)	0.294
Hand pain at activity (VAS)	7.23	7.55	0.32 (-0.58, 1.24)	0.480
Total number of IP nodes	7.91	8.59	0.67 (-0.86, 2.22)	0.384
Nº other sites with OA	1.25	1.41	0.16 (-0.35, 0.68)	0.534
PiS (kg)	1.91	1.66	-0.25 (-0.77, 0.27)	0.343
GrS (kg)	16.7	12.4	-4.34 (-7.19, -1.48)	0.003
Cochin	18.4	24.8	6.41 (-0.49, 13.3)	0.068
FIHOA	9.02	11.9	2.91 (0.06, 5.75)	0.045
Serum CRP (mg/dl)	0.32	0.44	0.12 (-0.21, 0.46)	0.465

CRP, C reactive protein; FIHOA, functional index for hand osteoarthritis; GSS, gray-scale synovitis; GrS, Grip strength; IP, interphalangeal; LIHOA, low income hand osteoarthritis; PiS, pinch strength; VAS, visual analogue scale

## Discussion

This is the first study reporting US data and clinical characteristics in a low-income cohort of patients with HOA. Our data reveal that inflammation, judged by the detection of a GSS or ST ≥ 2 in patients with symptomatic IP joints, was not associated with self-reported pain either at rest or activity. On the other hand, individuals with inflammation had worse functional capacity as reflected by a significant decrease in GrS and PiS in patients with higher GSS and ST values, revealed by a simple regression test analysis. There was no statistically significant difference when comparing results of both the FIHOA and Cochin questionnaires, which reflect compromise of functional status in patients with HOA, with US parameters. Data from the previous Nor-Hand study reported that isolated IP inflammation, also measured using GSS, was associated with pain in the previous 6 weeks and last 24 h prior to examination, as well as with more pain on palpation [13]. On the other hand, we did not find an association with pain and increased scores in GSS. This difference could have been due to the fact that we measured pain specifically in the most painful joint whereas data from the Nor-Hand study considered pain in all IP and carpometacarpal joints [13]. Those authors also reported an increase in GrS in patients with HOA affecting proximal or distal IP joints, as opposed to the decrease in GrS observed in our cohort. On the other hand, patients with carpometacarpal involvement in that study had a decrease in GrS. The authors claimed their result could be explained by the fact that individuals with an increased GrS have a higher chance of developing OA in the proximal IP joints. We may also consider that patients in the Nor-Hand cohort reported overall pain in the 24 h prior to evaluation whereas our patients were asked to score their pain both at rest and on movement in the last 48 h and we considered the most symptomatic IP joint for evaluation whereas the Nor-Hand study considered pain value in all IP joints. Our patients were apparently more symptomatic, given a mean pain level over 7 as compared to 3.2 in the Nor-Hand study [13]. Indeed, having pain above 4 in a visual analogue 0–10 cm scale was an inclusion criterion in our LIHOA cohort. Similar to the patients in the Nor-Hand study, we also had few patients with a positive PD sign as well as effusion although our numbers were higher. Remarkably, 6 (8.3%) out of our 72 included patients had a PD sign as compared to 23 (0.3%) of the individuals in the Nor-Hand study [13]. We can only speculate that the higher pain level and increase number of patients with PD sign in addition to considering the most symptomatic IP joint might well have influenced the decrease in GrS of our patients.

Data from a previous study with HOA patients conducted in the Netherlands reported a high association of inflammation based on synovitis detected with US and joint pain, functional impairment as well as quality of life assessed using the SF-36 questionnaire [26]. While we detected GSS ≥ 2 in less than 50% of our patients, GSS was reported in 96% of the patients in the Dutch study. However, the 8 (5–9) median pain value at movement in our patients compared to the median 4.9 (1-7.9) median pain in the Dutch study might indicate higher pain level in our patients [26] although we should remark that we addressed pain on movement whereas those authors did not specify this issue. Differences in methodology that limit comparison also include that we assessed the most symptomatic IP joint whereas those previous studies considered all IP joints. We should also mention that ours is a low-income cohort if compared to patients living in the Netherlands or in Norway [13, 16, 26]. This is reflected by the fact that over 41% of our patients had occupations classified as blue-collar jobs and only 33.3% had a college degree (data not shown). We have recently shown that social disparities were associated with worse function in our LIHOA cohort [16]. In the present study, although a higher GSS score was associated with lower GrS, used as a measure of functional impairment in HOA, GSS scores were similar regardless of occupations and income. Even considering that the study was not focused in these issues and may not have had statistical power to detect changes, we speculate that US alterations appear not to reflect changes related to socioeconomic issues in HOA patients.

Strengths of our study include being the first to report data from a low-income cohort. Patients had to fulfil the ACR criteria for HOA and no patient had other systemic arthropathy. The decision to exclude patients with fibromyalgia was due to the fact that pain evaluation could be influenced both by the concomitant disease as well as by concurrent medications. Patients were examined by a board-certified experienced radiologist that was not aware of clinical data, meaning blinding to level of disease severity, including radiological data. Pain level, as reported by the patient as well as GrS and PiS were registered by another blinded observer. We specifically addressed pain level both at rest and activity, since pain in patients with HOA is commonly associated with movement. Our study also has some limitations, which includes the relatively low number of individuals recruted, an issue that may have compromised statistical power to unravel differences among groups. The fact that our study was conducted during the pandemic affected inclusion of a larger number of patients. Indeed, 29 patients refused to undergo US examination because of social restrictions imposed at the pandemic period. Although some patients were taken medications, we believe that neither SYSADOA, which have not proved better than placebo in HOA, nor irregular and unchecked use of NSAIDs or low-dose prednisone had any clinical relevance to our present results [10].

In conclusion, clinical and imaging data of patients from a low-income cohort mimick results reported in wealthier regions. Inflammation judged by US parameters was associated to lower GrS and PiS but not pain level in patients with symptomatic IP HOA.

## Data Availability

All data generated or analysed during this study are included in this published article.

## Abbreviations

ACR: American College of Rheumatology
BMI: Body mass index
CRP: C reactive protein
DIP: Distal interphalangeal
FIHOA: Functional Index for HOA
GrS: Grip strength
GSS: Grayscale synovitis
Hand OA: Hand osteoarthritis
IP: Interphalangeal
JE: Joint effusion
K-L: Kellgren-Lawrence
LIHOA: Low income HOA
NSAIDs: Nonsteroidal anti-inflammatory drugs
OA: Osteoarthritis
OMERACT: Outcome Measures in Rheumatology
PD: Power Doppler
PIP: Proximal interphalangeal
PiS: Pinch strength
ST: Synovial thickening
SYSADOA: Symptomatic slow acting drugs for OA
VAS: Visual analogue scale
